# Snowfall Variability Dictates Glacier Mass Balance Variability in Himalaya-Karakoram

**DOI:** 10.1038/s41598-019-54553-9

**Published:** 2019-12-03

**Authors:** Pankaj Kumar, Md. Saquib Saharwardi, Argha Banerjee, Mohd. Farooq Azam, Aditya Kumar Dubey, Raghu Murtugudde

**Affiliations:** 1 Earth and Environmental Sciences, Indian Institute of Science Education and Research Bhopal, Bhopal, 462066 India; 2 Earth Climate Sciences, Indian Institute of Science Education and Research Pune, Pune, 411008 India; 3 Discipline of Civil Engineering, Indian Institute of Technology Indore, Indore, 453552 India; 4 University of Maryland, College Park, USA

**Keywords:** Climate and Earth system modelling, Cryospheric science

## Abstract

Glaciers in the Himalaya-Karakoram (HK) are critical for ensuring water-security of a large fraction of world’s population that is vulnerable to climate impacts. However, the sensitivity of HK glaciers to changes in meteorological forcing remains largely unknown. We analyzed modelled interannual variability of mass balance (MB) that is validated against available observations, to quantify the sensitivity of MB to meteorological factors over the HK. Within the model, snowfall variability (0.06 m/yr) explains ~60% of the MB variability (0.28 m/yr), implying a sensitivity of MB on snowfall to the tune of several hundreds of percent. This stunningly high sensitivity of MB to snowfall offers crucial insights into the mechanism of the recent divergent glacier response over the HK. Our findings underscore the need for sustained measurements and model representations of the spatiotemporal variability of snowfall, one of the least-studied factors over the glacierized HK, for capturing the large-scale and yet region-specific glacier changes taking place over the HK.

## Introduction

The Himalaya-Karakoram (HK), covering a glacierized area of ~41,000 km^2^ is one of the largest mountain ranges on Earth^1^. HK is surrounded by densely populated countries of south Asia and more than 800 million people depend strongly on water originating in its river systems (Indus, Ganges and Brahmaputra) for drinking, irrigation, hydropower, and industrial purposes^2,3^. Given the intrinsic variability of regional precipitation fed by Western Disturbances (WDs) and Indian Summer Monsoon (ISM), HK glaciers buffer a significant fraction of the population against droughts^3^. Climate change is threatening the future of glaciers in the HK^4^, putting this dynamic water reserve under severe stress and causing serious hydrological changes^3,5,6^. This is likely to expose a significant fraction of regional population to serious climate impacts^3,7,8^. Understanding the details of the climatic forcing that is driving the glacier changes over the HK, therefore becomes an imperative for future adaptation and mitigation strategies.

The glaciers over Karakoram are mostly fed by snowfall occurring during winter months (November to April) derived from WDs while glaciers in the Himalaya receive snowfall from both WDs and ISM^9^. Region-wide remote-sensing glacier mass balance (MB) estimates have revealed that HK glaciers have mostly been experiencing a mass loss in recent decades^10,11^. Available field data from a handful of Himalayan glaciers also confirm this fact^12,13^. However, the pattern of the mass loss is quite heterogeneous; glaciers in the Himalaya are losing mass at rates that vary locally^11,12^ while Karakoram glaciers have been in a near mass-balanced state over the past few decades^10,14,15^. The anomalous behavior, initially termed as “Karakoram Anomaly”^16,17^, is now found to be centered in Kun Lun Shan^10^. The heterogeneous mass balance behavior over HK points to local idiosyncrasies of net climate forcing and glacier response that await a clear explanation.

Due to the harsh field conditions, only a few studies have been attempted to understand the meteorological forcing of glacier MB in the HK using *in-situ* data^18,19^. Relevant meteorological field-data from the glacierized high mountains of the region are quite limited^20,21^. A few model studies have investigated the regional glacier MB patterns utilizing various interpolated/reanalyzed meteorological data products^21–24^. A recent study^25^ elucidated the importance of local MB sensitivity to temperature in determining glacier response. Another study using station data over the Karakoram and Tibetan Plateau suggested that cooling summertime surface air temperatures induce less glacier melting over the Karakoram. This cooling is further linked with the “Karakoram Vortex” under climate warming scenarios^26,27^. The vortex modulates the air temperature over the western Tibetan Plateau mainly through adiabatic sinking/rising processes^28^. However, the role of other meteorological parameters in driving the observed spatiotemporal pattern of glacier mass loss in the HK remains largely unexplored. A clear answer to the questions about drivers of HK glaciers in a warming world is needed to guide future developments related to measurement and modelling strategies to advance process and predictive understanding, and to deliver reliable projections for this dynamic storage of water.

Glacier MB variability is a net result of climatic forcing, which consists of surface MB and a relatively slow response of ice flow^29^. While the long-term MB trends reflect the combined effects of these, the short-term interannual fluctuations of MB are mostly due to the meteorological forcing. Therefore, the correlations between annual time series of glacier MB and the meteorological variables encode the sensitivity of the former to changes in the latter. In fact, the interannual variability of glacier MB is rather large relative to the recent long-term net MB especially in the Himalaya^12^. It is also expected that some of the drivers themselves vary coherently due to mutual feedbacks.

Glacier surface MB is determined by a balance between accumulation and ablation, with the ablation being controlled by the net incoming energy flux at the surface. The major contributor to accumulation is snowfall (SF), while the surface energy balance is mostly determined by the balance of short-wave (SW) and long-wave (LW) radiation, sensible heat flux (SHF) exchanged with the atmosphere, and latent heat flux (LHF) due to moisture exchange with the atmosphere via sublimation and condensation,

1 $$MB=SF-(SW+LW+SHF+LHF)/\rho L+{\rm{\varepsilon }}{\rm{.}}$$

Where *ρ* density of ice, L is is latent heat of melting, and ε denotes relatively minor contributions to this balance; e.g., that due to refreezing of melt-water, conductive heat flux, geothermal and frictional heating, etc^30^.

The primary objective of the present study is to identify the major meteorological drivers of MB fluctuation over the HK region. We first discuss the performance of the model in capturing the variability of MB from regional to individual glacier scale. Then we analyze the drivers of the modelled interannual MB variability within the model assumptions. The implications of the results are discussed in the context of the ‘Karakoram anomaly’.

## Results and Discussion

### Modelled mass balances

**Figure 1:**
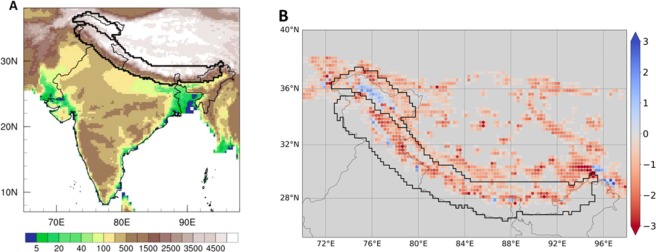
Spatial pattern of mass balance. (**A**) The model domain with topography (meter). (**B**) The modelled MB (m.w.e./yr) pattern over the simulated domain, for the period 1989–2016.

A previous study^24^ established that REgional MOdel (REMO) coupled with a dynamic glacier scheme (REMO_glacier_) is capable of reproducing the general pattern of decadal-scale glacier mass changes in the High Mountain Asia, including the Karakoram Anomaly^16,17^. In the present study, annual MBs over the HK region during 1989–2016 are simulated using REMO_glacier_ for the glacierized fraction of each grid cell (Fig. 1B). REMO_glacier_ simulates a negative MB over most parts of the region except the Karakoram. These positive MBs over the Karakoram and Kunlun mountain range have been addressed by several studies^24,31^. In this study, the modelled mean glacier MBs were estimated to be −0.74 m.w.e./yr for the Himalaya and 0.06 m.w.e./yr for the Karakoram between 1989 and 2016 (Table S2). The estimated value for the Himalaya suggests a negative bias in the model result. However, the model reproduces the general pattern of glacier mass loss in the HK (Fig. 1). There are some isolated gridboxes with positive MB values (Fig. 1B) that are not consistent with observed glacier MB. These are likely to be model artifacts related to steep topographic gradients between neighboring gridboxes^24^.

### Region-wide validation of modelled mass balances

**Figure 2:**
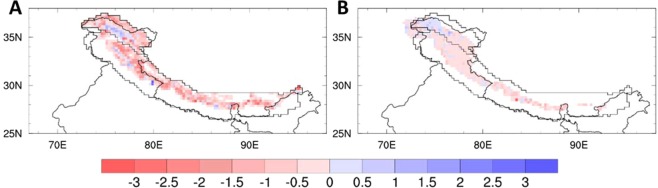
Comparison of REMO_glacier_ with geodetic data (**A**) REMO_glacier_ simulated mean MB (m.w.e./yr) comparison with (**B**) Geodetic MB calculated (for details ref. ^11^) (m.w.e./yr) for the period 2000–2016. For model, all the glacier fraction gridboxes covering the data is represented.

We compared the modelled MBs with the latest remotely sensed geodetic MB estimates across the HK for the period 2000–2016^11^. There is a reasonable match between these two datasets at regional to sub-regional level (Fig. 2, Table 1), though model estimates are systematically more negative. There is a large variability in both the modelled and geodetic estimates over gridboxes with a small glacier fraction added to the spatial noise. Also, as discussed in ref. ^24^, the lack of a description of the large-scale flow within model gridboxes may exaggerate the snowfall variability from one gridbox to another, leading to an inflated spatial variability of modelled MB. This limitation is discussed in detail in a previous study (for details see ref. ^24^). Despite the model limitations, the general consistency model data with observation as described above indicates the overall reliability of the modelled regional MB.

**Table 1:** Validation of modelled mass balances with available mass balances (m.w.e./yr) for Himalaya, Karakoram and Chhota Shigri (CSG), Mera and Pokalde Glaciers over different time periods.

Regions	Measurement	Period (yr)	Modelled (m.w.e./yr)	Observed (m.w.e./yr)
Himalaya	Geodetic	2000–2016	−0.72 ± 0.05	−0.39 ± 0.02
Karakoram	Geodetic	2000–2016	−0.09 ± 0.09	−0.05 ± 0.03
HK	Geodetic	2000–2016	−0.39 ± 0.06	−0.28 ± 0.03
CSG	Field	2003–2014	−0.12 ± 0.10	−0.56 ± 0.18
Mera	Field	2008–2015	−0.03 ± 0.14	−0.02 ± 0.15
Pokalde	Field	2010–2015	−0.006 ± 0.15	−0.69 ± 0.20
Himalaya (pentad*)	Field	1990–2014	−0.76 ± 0.05	−0.72 ± 0.09

*Pentad observation is the mean of 24 glaciers where some glaciological MB data is available.

**Figure 3:**
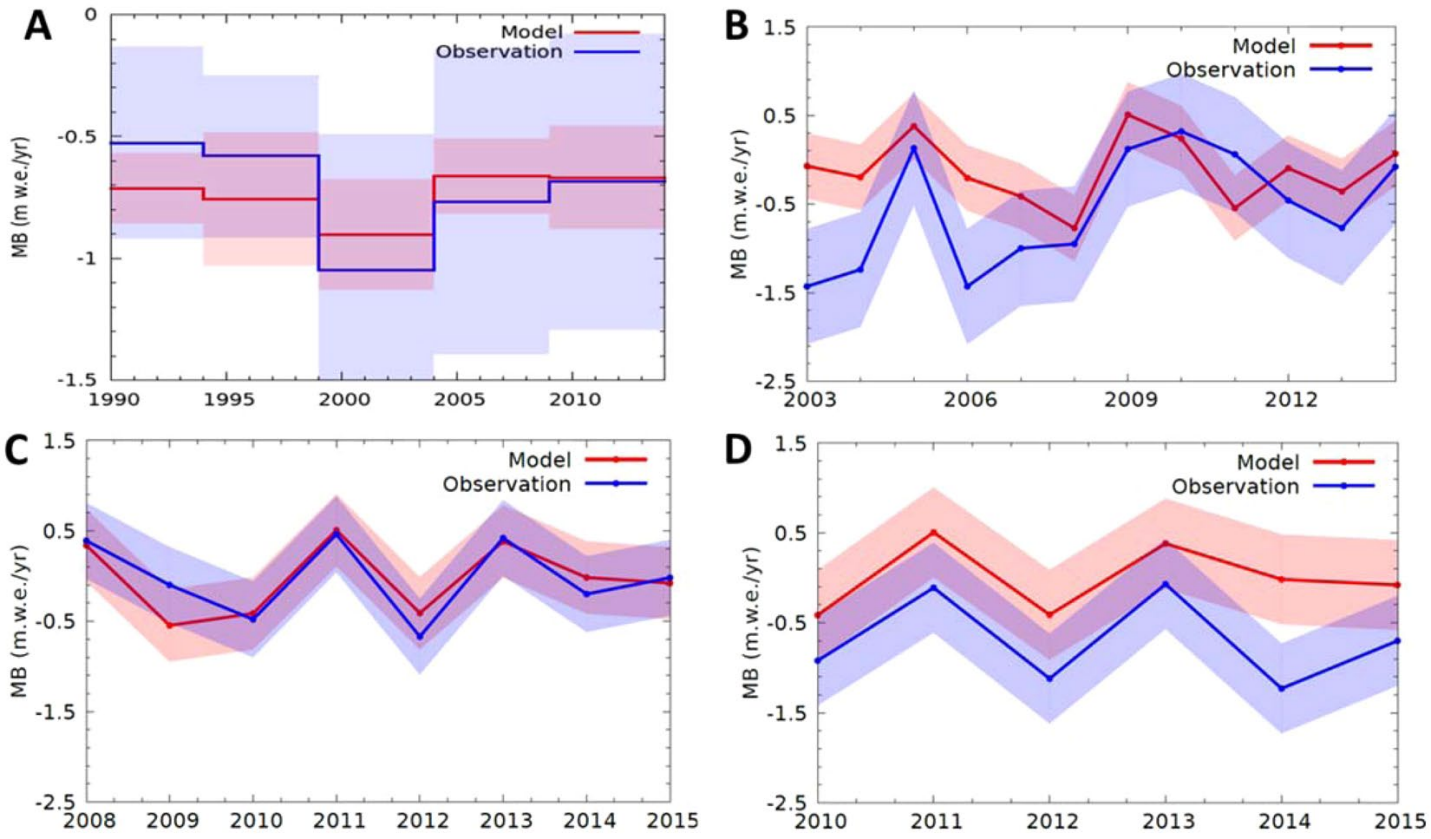
Mass-balance variability in the Himalaya. (**A**) A comparison of pentadal averages of modelled annual MBs (red line) with that of pentadal averages from all the available field observations of MBs (blue line) from 24 glaciers over 1990–2014 period^12^. (**B**) A comparison of modelled annual MB (red line) with that of available field annual MBs (blue line) on Chhota Shigri Glacier over 2003–2014^18^, (**C**) Mera Glacier for period 2008–2015^18^ (**D**) Pokalde Glacier for 2010–2015^18^. Corresponding 1 - σ bands are also shown.

We also validated the simulated MBs over the Himalaya with pentadal (5 year) averages of all the available glaciological field measurements of MB from 24 glaciers in the Himalayan region^12^ (Fig. 3A). Here, the observed pentadal MBs are the averages of all the available field measurements of glacier MB for the corresponding pentad from the entire Himalaya during 1990 to 2014. The model captures the observed pentadal average MBs from the Himalaya reasonably well (Table 1), though the standard deviation of modelled pentadal MB is relatively smaller (Fig. 3A). We note that a possible negative bias in the observed MB due to avalanches and other effects was pointed out in MB series of some of these glaciers^12,32^ making gridbox scale comparison difficult. Regrettably, no annual glaciological MBs from any of the Karakoram glaciers are available^12^.

### Glacier-wide validation of modelled mass balances

Chhota Shigri Glacier has the longest observed MB time series in the whole Himalaya since 2003^18^. For glacier-wide model validation, we simulated the annual MBs on Chhota Shigri Glacier and compared them with observed MBs for the period 2003–2014 (Fig. 3B). The model is reasonably good at capturing the individual glacier mass balances (Table 1) and interannual variability (Fig. 3B), corresponding correlation (0.53) is significant at ~95% confidence level. Additionally, model results were compared with MB data^19^ from two more glaciers in the Himalaya, namely, Mera (Fig. 3C) and Pokalde glaciers (Fig. 3D) for relatively shorter periods of 8 and 6 years, respectively. Interestingly, the interannual variability of MB was also well captured for Mera and Pokalde glaciers (Fig. 3C,D) with significant correlations. However, it must be noted that given the coarse resolution, and a simplified description of glacier dynamics and geometry, it is not expected that the model would be able to reproduce glacier-scale MB accurately for any given glacier over a particular balance year. For example, the positive trend of the observed annual MBs for Chhota Shigri is not captured well in the corresponding modelled time series, and there is a clear positive bias in the modelled MBs for Chhota Shigri and Mera glaciers (Table 1).

Overall, the model may not reproduce local-scale glacier-specific MBs accurately due to inherent model limitations and has a negative bias in the case of regional-scale MBs. However, it does a reasonable job of reproducing the general spatial pattern of mean multi-decadal MB (Figs. 1,2), the typical magnitudes of regional MBs (Table S1), and pentadal to annual-scale variability (Fig. 3).

Apart from glacier-wide MB, we have also compared the annual snowfall in four western Himalayan stations for which we could access the data. The model seems to be able to capture the observed interannual variability and magnitude of snowfall at these stations reasonably well (see Supplementary Fig. S1). A reasonable model performance in reproducing observed precipitation and temperature has already been established in ref. ^24^.

### Fluctuations of modelled mass balance and its drivers

**Figure 4:**
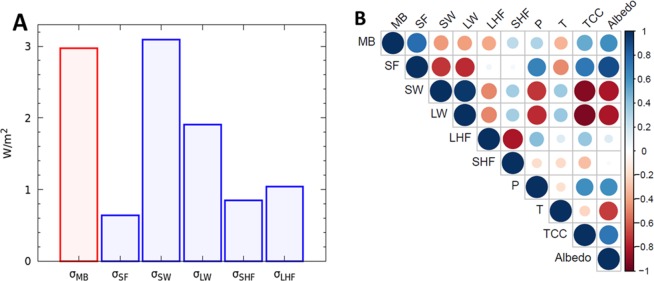
The variability of MB and its drivers. (**A**) The variance (σ) of annual MB (red bar), and that of the dominant mass/energy-balance terms (blue bars) that contribute to it, viz., annual snowfall (SF), net short-wave energy (SW), net long-wave energy (LW), sensible heat flux (SHF), latent heat flux (LHF). All mass terms are expressed in equivalent energy units (W/m^2^) for ease of comparison. (**B**) A graphical representation of correlations among the interannual variability of glacier MB in the HK and its meteorological drivers during 1990–2016. The dominant control of SF variability over that of MB fluctuation is evident.

Our analysis of the 28-year-long time series of modelled MBs and the associated meteorological parameters over the HK revealed that SW has the largest interannual variability, followed by MB, LW, LHF and SHF, respectively (Fig. 4A). Notably, among the parameters considered, SF has the smallest variability in absolute terms. If these fluctuations were to be uncorrelated, the expected variance of annual MB of the HK glaciers would be obtained by combining the variance of the drivers in quadrature, which turns out to be about 4 W/m^2^. However, the observed interannual MB variance is equivalent to only about 3 W/m^2^ (Fig. 4A). This suggests that there are strong correlations among the drivers. Thus, the net effect of a specific driver on MB, despite having a large variance, may be small due to the cancelling effects of other drivers. In contrast, a positive correlation among drivers could magnify the effect of the ones with a relatively weak variability. To untangle such cancelling/enhancing effects on MB, we analyzed correlations between all the possible pairs of fluxes listed above and MB. In addition, annual precipitation (P), temperature (T), total cloud cover (TCC), and albedo were considered. The results are shown in Fig. 4B (also see Supplementary Fig. S2, see Supplementary Fig. S3) and discussed in the next section.

### Snowfall variability as the major driver for mass balances

The correlation matrix (Fig. 4B and Supplementary Fig. S3) reveals that SF variability has the strongest correlation with the MB response, with a correlation coefficient (CC) of 0.76 (p < 0.001) within our model assumptions. It is quite surprising that the interannual MB variability of 0.28 m.w.e./yr in the HK is so tightly controlled by the interannual variability of SF that has a relatively small magnitude of only 0.06 m.w.e./yr. This implies a strong MB sensitivity of ~470% to changes in SF in the HK. The reason behind this is that SF not only contributes directly to the accumulation, but it also has a strong effect on net budget of SW (CC = −0.72, p < 0.001) through its control on albedo (CC = 0.88, p < 0.001) and association with TCC (CC = 0.71, p < 0.001). Both albedo and TCC, with their respective variability of 5% and 8% within the model, exert a strong influence on the radiation budget in the HK. Thus, cooperation among the above factors amplifies the net effect of SF variability on MB, resulting in a very large sensitivity of MB to changes in SF.

**Figure 5:**
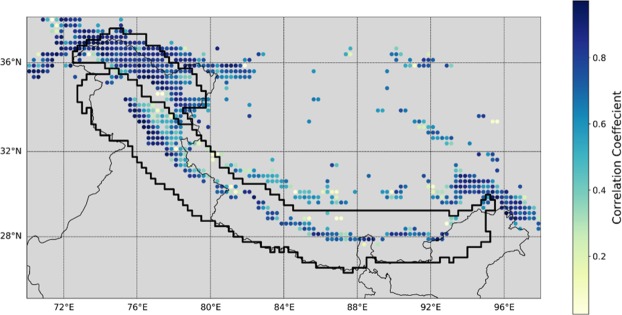
SF correlation with MB (1990–2016). MB and SF over the HK have a strong correspondence, revealing the control of the variability of SF on that of MB.

The correlation analysis also reveals that the role of SF is stronger in the Karakoram than in the Himalaya (see Supplementary Fig. S4). The surprisingly dominant role of SF in controlling MB is also verified with a principal-component analysis (PCA) of the correlation matrix (see Supplementary Fig. S5). This critical role of the SF variability as a MB driver is, in fact, evident from a simple and yet striking plot of both the time series (see Supplementary Fig. S4) and spatial correlation of SF and MB (Fig. 5). The CC is high over the Karakoram and parts of Eastern Himalaya where glacier-fraction is relatively high. However, its value over the Western Himalaya is lower than other regions because glacier fraction is quite low (<10%; see Supplementary Fig. S6). Hence small changes in magnitude may show a relatively large variability. Regions with higher (lower) SF have significant (insignificant) correlations between SF and MB, which is seen in both the model and ERA-Interim reanalysis (ERAI) data (see Supplementary Fig. S7). The spatial correlation between these two datasets also depicts the same pattern (see Supplementary Fig. S7). This analysis provides further evidence that indeed SF has a strong relationship with MB over the whole region of study, and that this result is not a model artifact.

We note that instead of mean annual temperature, using summer (JJA) temperatures improves the correlation with MB (see Supplementary Fig. S8). However, this does not necessarily imply a stronger control of SHF on MB, as the net radiation budget is known to play a dominant role in controlling glacier melt^33^. The stronger correlation with summer temperature is expected as temperature is a good indicator of net local energy budget^34^.

### Relative insensitivity of MB in the karakoram to temperature change

Our model results suggest similar warming trends in both the Himalaya and the Karakoram, with a rate of temperature change of about 0.3 °C per decade (p < 0.1) (see Supplementary Fig. S9). While no precipitation trends are seen in either of the regions for the study period as per the model results, any local warming can influence the partitioning of total precipitation into snow and rain, leading to a reduction in SF. The same insignificant changes in precipitation have been reported earlier over the Karakoram region^35,36^. Indeed, the above warming is accompanied by a statistically significant 0.08 mm/day per decade reduction of SF in the Himalaya (p < 0.1) (see Supplementary Fig. S9). However, SF has not changed significantly in the Karakoram during this period (see Supplementary Fig. S9) suggesting a lower sensitivity of SF over the Karakoram to warming. This is consistent with the argument^22^ that a dominant wintertime snowfall in the cold Karakoram has been insensitive to local warming thus far. We note that, alternatively, the variability of a large-scale circulation system, the so-called “Karakoram Vortex”, has been argued to have led to a recent summertime cooling over the Karakoram^26,27^. This alternative explanation of the Karakoram anomaly does not rely on the above-mentioned insensitivity of glacier mass balance to annual temperature variability in the Karakoram. However, these results do not rule out that a dominant wintertime SF in Karakoram is less prone to conversion into rain as argued by ref. ^22^ and supported by our model results.

In contrast to the situation in the Karakoram, a monsoon-derived summer snowfall in the Himalaya is negatively affected by local warming, causing more rainfall at the expense of solid precipitation. Thus, strongly monsoon-fed Himalayan glaciers have a higher sensitivity of MB to temperature change. This, together with the strong control of SF on MB described above, lead to a strongly negative mass budget for the Himalayan glaciers within the model.

The above contrast in the response of SF to local temperature changes is likely to be one of the root causes for the divergent behavior of glaciers in the Himalaya and Karakoram in a warming climate. The same inference can be deduced from the fact that there is no significant correlation between local interannual temperature fluctuation and SF in the Karakoram, but in the Himalaya the two are locally anti-correlated (CC = −0.65, p < 0.001) (see Supplementary Fig. S10) within our model assumptions.

## Summary and Conclusion

The annual MBs of the HK region are simulated using REMO_glacier_ over the 1989–2016 period. The spatial patterns of simulated MB show an overall negative mass balance but also reveal regions with positive mass balance anomalies especially over the Karakoram. This positive MB is also highlighted in previous studies. However, there is an overall negative bias in our modelled MB that may likely be related to biases in atmospheric forcing during the simulations or due to the effects of unresolved topography and relief, and unaccounted for local circulation effects. For the whole HK region, it has been found that MB simulated by model is able to capture observational variability reasonably well. An analysis of the model data reveals that the annual glacier MB variability in the HK over the last two and half decades is essentially driven by the variability of mean annual snowfall. We also show that, within the model, a relative insensitivity of snowfall to the local temperature changes are responsible for the Karakoram anomaly, while the negative impact of climate warming on monsoon-derived summer snowfall in the Himalaya forces a net negative MB in the region. It is thus apparent that understanding the recent and future climate forcing and the corresponding response of the glaciers at the third pole would require a strong handle on snowfall variability and its trend. For example, can the winter warming cross the threshold beyond which winter snowfall would be affected significantly even in the Karakoram? Unfortunately, snowfall is one of the biggest unknowns in the region, with very scant or no long-term field data^20,37^. While snow-cover extent and albedo are accessible through remote-sensing measurements, the snow-water-equivalent - which is crucial for any glaciological or hydrological mass-budget considerations - is difficult to constrain from satellite data^38,39^. There has been a spate of scientific activities over the past decade or so, as far as remote-sensing and field-based observations, and modelling of glacier dynamics in the HK are concerned. This has led to a significant process understanding of the response of HK glaciers and their role in the regional water cycle. Our results indicate that snowfall needs to be targeted more specifically, if the efforts to piece together a complete picture of the water-cycle in the glacierized HK are to succeed. The broader impact of our results can hardly be overemphasized in terms of the need to understand the glacier-specific extraction of seemingly weak climate links to understand the net impact of global warming on glacier mass budgets.

## Data and Methods

### Regional model REMO

The REgional MOdel (REMO) is a regional climate model^40,41^ which derives its dynamic core from the Europa Model of the German weather service^42^. It uses a rotated grid with the equator in the middle of the model domain to avoid disproportionate grid sizes towards the poles. The horizontal coordinates are discretized on the Arakawa C-grid, and there are 27 vertical hybrid levels^43^. Time discretization follows the leapfrog time stepping with semi-implicit correction and Asselin filter smoothing. The prognostic variables of REMO are surface pressure, temperature, horizontal wind components, water vapour and cloud water content. The model uses a fourth order linear horizontal diffusion of momentum, temperature and water content^44^. A sponge zone of 8 gridboxes exists at the boundary of the model domain^45^ where the lateral boundary conditions are applied for the aforementioned prognostic variables.

The physical parameterizations for REMO are adopted from the global climate models ECHAM4 and 5^46,47^. Soil heat transfer is based on a 5-layer model to a depth of 10 m where heat flux is set to zero at the bottom. Water budget equations are solved for the three reservoirs: soil moisture, vegetation (interception reservoir) and snow while the runoff scheme is based on^48^ which considers sub-grid scale variations of field capacity over the inhomogeneous terrain. A radiative upper boundary condition following^49,50^ is applied. Radiation (SW and LW) is parameterized^51^ with modifications for ECHAM4 provided^47^.

### Glacier scheme

A dynamic glacier scheme (DGS) is developed and implemented in REMO. DGS has a unique ability to simulate the MB and dynamically adjusted glacier-surface fraction of each gridbox depending on the accumulation and ablation conditions^52^. To augment DGS online with REMO, in addition to the standard REMO gridbox fractions mentioned above, a fourth fraction for glaciers has been introduced^52^. REMO with this fourth glacier fraction is hereafter referred to as REMO_glacier_. A glacier fraction in a REMO_glacier_ gridbox includes the total area covered by glaciers in that gridbox. Glaciers are allowed to grow and shrink according to their MB, but they are restricted to the land fraction of a single model gridbox and are not allowed to expand over water. Individual glaciers in a single gridbox are represented by a two-layered cuboid of surface area A and thickness h, corresponding to a volume V. Since glacier thickness has been shown to depend on glacier area^53,54^, glacier area is used to calculate volume from a power law relation, V = cAγ where c and γ are empirical constants^55^.

The surface energy balance is calculated at each time step for the glacierized fraction according to the following equation:

2 $${\rm{dQice}}/{\rm{snow}}={\rm{SW}}+{\rm{LW}}+{\rm{SHF}}+{\rm{LHF}}+{\rm{G}}+{\rm{M}}$$

where G is ground heat flux, M is amount of energy consumed during melt of snow and ice, and dQice/snow is the energy change of heat content in the upper snow or ice layer.

### Experimental setup and analysis

REMO_glacier_ is integrated over South-Asia (Fig. 1A) for the period 1989–2016 at a horizontal resolution of 0.22° × 0.22° (~25 km) with 27 vertical levels and forced by the ERA-Interim reanalysis (ERAI) dataset^56^ as the lateral boundary conditions. The update of lateral boundaries has a temporal resolution of 6 hours and is interpolated into a two-minute time step. The glacier volume data^57,58^ is used to initialize REMO grid-cells. The REMO_glacier_ simulation is done for entire South-Asia while results are only shown for Karakoram (K) and Himalayan (H) regions (as defined in ref. ^1^).

### Statistical analysis

The analyses focus on simulated annual (1 October to 30 September of next year) mean statistics of various model parameters and are compared against the available observations. Different statistical methods have been used to determine correlations, trends, and significance analysis. The standard Pearson-correlation method has been used to determine the relationship between variables, while student t-test is used to assess the significance of the correlation. Mann-Kendall test is a well defined method to determine the non-parametric monotonic trend in the dataset with the significance level^59–61^. We used this test to determine the trend of MB as well as other parameters to check whether statistical significance changes with time. Principle component analysis (PCA) is the multivariate analysis method to determine the degree of relationship among variables for redundancy reduction^62,63^. We used PCA to describe the variable that is the best estimate of MB variability over the study region.

### Observational datasets employed

The model-simulated results are compared with different observational data sets. The ERAI^56^ and ERA5^64^ reanalysis products, along with Indian Meteorological Department (IMD) station data from four western Himalayan stations are used for comparison of SF with modelled MB. The geodetic pentadal dataset of MB^12^ over the whole Himalaya is used to validate the model for the period 1990–2014. The glaciological mass balance for the period 2003–2014 over Chhota Shigri glacier, for the period 2010–2015 for Pokalde glacier, and 2008–2015 for Mera glacier^18^ are further used to assess the performance of the model to capture individual glaciers’ MB values. To cover the whole HK region, ASTER geodetic MB data at 0.25° × 0.25° is used^11^ for the period 2000–2016.

## Supplementary information

SUPPLEMENTARY File

## Data Availability

Datasets are given in the supplementary Information. The additional dataset that support the findings of this study are available from the corresponding author on reasonable request.
