# Supplementary material for: Snowfall Variability Dictates Glacier Mass Balance Variability in Himalaya-Karakoram
Source: Sci Rep. 2019 Dec 3;9:18192. doi: 10.1038/s41598-019-54553-9 (PMC6890783; doi:10.1038/s41598-019-54553-9)
Supplement: Supplementary file 1 — SUPPLEMENTARY File [file 41598_2019_54553_MOESM1_ESM.docx]

**Supporting Information**

**Snowfall Variability Dictates Glacier Mass Balance Variability in Himalaya-Karakoram**

**Pankaj Kumar^1^, Md. Saquib Saharwardi^1^, Argha Banerjee^2^, Mohd. Farooq Azam^3^,**

**Aditya Kumar Dubey^1^ and Raghu Murtugudde^4^**

^1^Earth and Environmental Sciences, Indian Institute of Science Education Research Bhopal, Bhopal 462066, India

^2^Earth Climate Sciences, Indian Institute of Science Education Research Bhopal Pune, Pune 411008, India

^3^Discipline of Civil Engineering, Indian Institute of Technology Indore, Indore 453552, India

^4^University of Maryland, USA

Corresponding author: Pankaj Kumar (kumarp@iiserb.ac.in) & Argha Banerjee (argha@iiserpune.ac.in)

**Contents of this file**

Figures S1 to S11

Tables S1 to S2

**Model and Observational Snowfall**- Figure S1 shows the time series of station and modelled SF along with their correlation coefficients over the four stations. Interestingly, modelled SF showed good skill in capturing the SF variability and trends at all stations including reanalysis data (ERA5). Magnitude bias in some stations are apparent and is expected as we are comparing point observations with a ~625 km^2^ area, which is very challenging for a model.

Figure S6 shows SF over the region having glacier fraction less than 10%. Figure S7-A shows the mean value of simulated and reanalysis SF over glacierized gridboxes of the HK region, while their spatial correlation is shown in Figure S7-B. The Model is able to reproduce the SF magnitude for the Karakoram as well as for the eastern Himalaya. Less glaciated gridbox fractions of WH showed a lower correlation with SF.

**MB drivers and their relationship:**

Figure S2 shows a pictorial representation of the different parameters observed in the study. Red (Blue) shows the drivers for negative (positive) MB, while temperature has both effects irrespective of the seasons. The statistical relationship between these parameters is shown in Figure S3 for the HK region, while Figure S10 and Figure S11 show the H and K regions, respectively. All analysis has been done at the annual scale, i.e., 1 October to 30 September. We find that annual SF fluctuation has the strongest correlations (0.76, p < 0.001) with MB fluctuations. The next most important factors of SW and LW have much weaker roles to play with correlation coefficients of -0.43 (p<0.1), and -0.42 (p<0.1) respectively (Figure S3). The correlation matrix also reveals the strong effect of interannual SF fluctuations despite its relatively small variance. The SF variability not only contributes to the accumulation directly but also has a strong effect on SW (- 0.72, p < 0.001) and LW (- 0.75, p < 0.001), through its control on albedo (0.88, p < 0.001). The SF, in turn, is strongly affected by fluctuations of P and TCC. A Principal Component Analysis (PCA) of the correlations also supports this inference of a dominating role of SF fluctuation as far as mass balance variability is concerned. For simplicity, we only consider the fluctuations of MB, SF, SW, T, and LHF for the PCA. We ignore LW and SHF due to their strong correlations with SW and SHF, respectively.

To understand the relationship between the most robust parameter, i.e., SF with MB, we have shown the inter-annual variability of their relationship for all regions (Figure S4). These two parameters have the highest correlation over K compared to HK, followed by H. The data inter-relationship indicates that many variables are interlinked. Therefore, covariability can be best determined by PCA. The scree plot (Figure S5-B) shows that two initial PCs explains more than 80% of correlation of the variables MB, SW, SF, and LHF. Biplot (Figure S5-A) also indicates the most robust relationship between MB and SF followed by other parameters. MB and SW have correlations with both the principal components, with stronger overlap with F1. Thus, F1 represents a robust control of SW fluctuations on MB variability, and on the corresponding variations of SW through albedo feedback. This reinforces the inference drawn from the correlation plot (Figure S5-A).

**MB biases and interannual variability**- MB is compared with observational geodetic data (Brun et al., 2017) over different regions identified by Brun et al., (2017). The Model is able to capture the mean MB with varying scales of biases (Table S1). Biases over East Nepal, Nyainqentanglha and Karakoram, lie in the range of ±10 m.w.e./yr while other regions are showing relatively higher biases. The annual values of MB over HK, H and K are shown in Table S2. The trend of MB over HK and K shows a decreasing trend for the whole period.


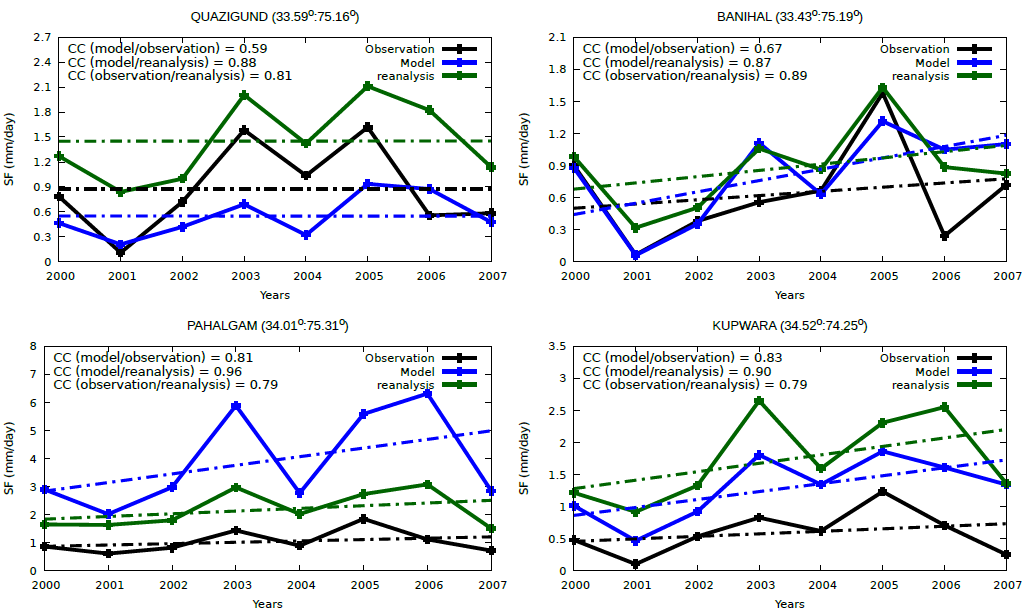


**Figure S1:** Interannual variability of snowfall over four stations of Western Himalaya. Observation station data is obtained from the Indian Meteorological Department, while reanalysis data is the latest ECMWF product (ERA5). The model takes the mean of the surrounding gridboxes for calculation over a station.


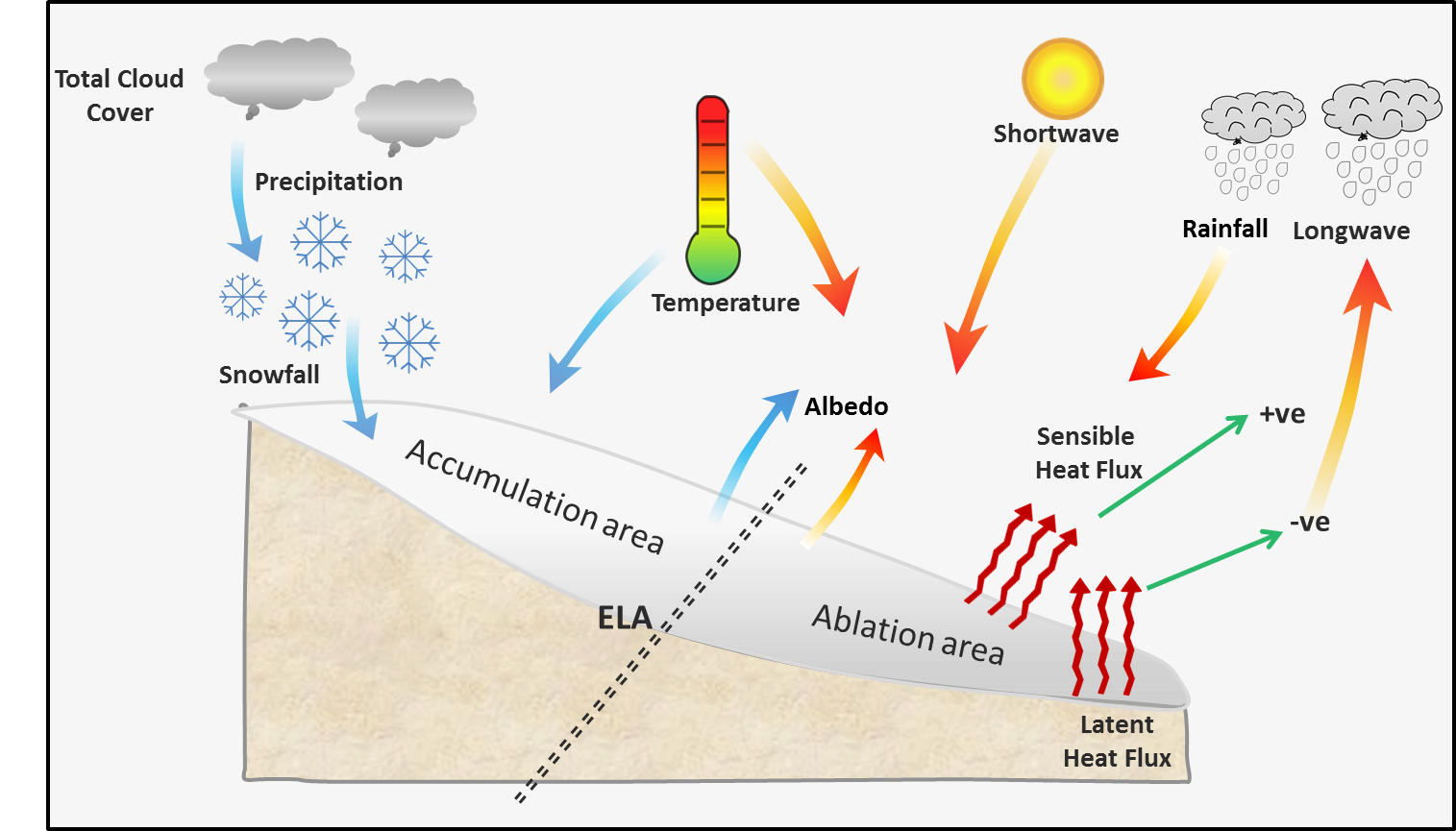


**Figure S2. Pictorial representation of Factors affecting MB.** Blue shows positive contributors of MB and red shows negative contributors of MB.

**
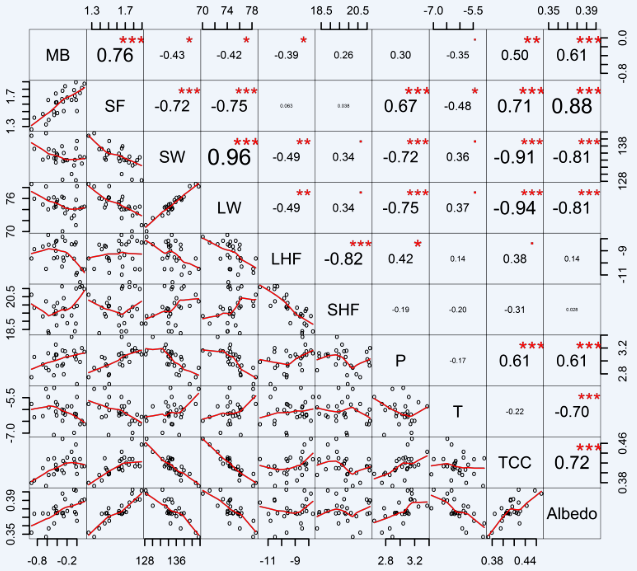
**

**Figure S3. Correlations Matrix. The correlation matrix for MB and its drivers over the HK** (A numerical representation of Fig. S2 and 4B). MB has units m.w.e./yr, SF is in mm/day, and energy terms are in unit W/m^2^. Total cloud cover and albedo are fractions in the range 0-1.0.


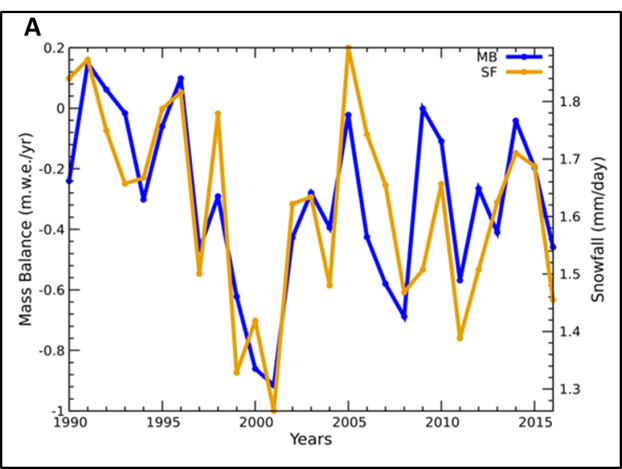

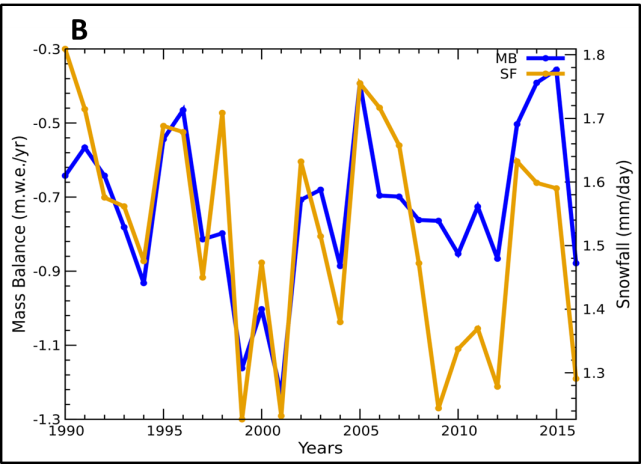


**
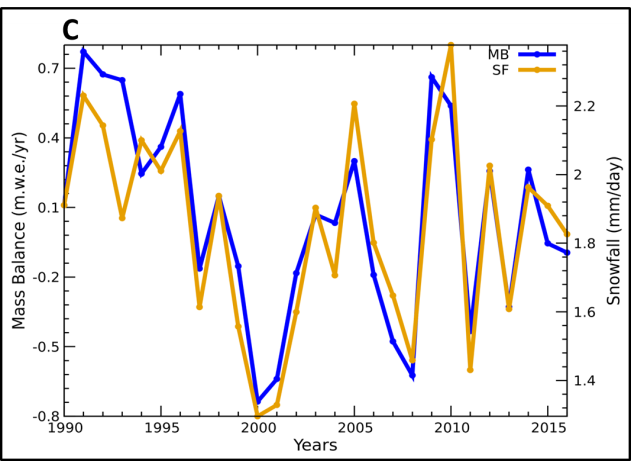
**

**Figure S4. SF and MB variability in the HK, Himalaya (H) and Karakoram (K). A)** Time series of annual MB and SF in the HK.  **B)** Time series of annual MB and SF in the Himalaya. **C)** Time series of annual MB and SF in the Karakoram. The two quantities have a stronger correlation in the Karakoram than in the Himalaya. This figure shows that MB has the higher correlation with SF over K compared to H region.

**
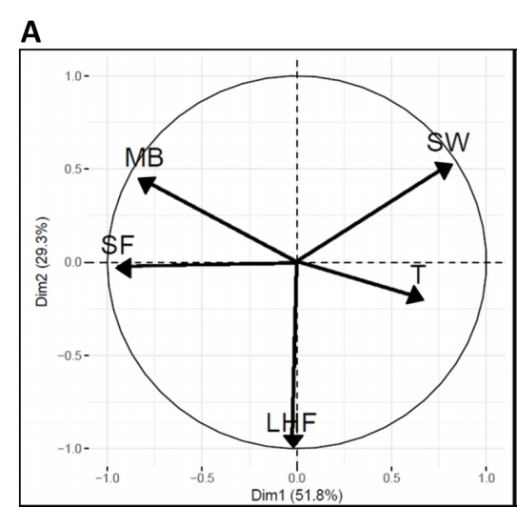

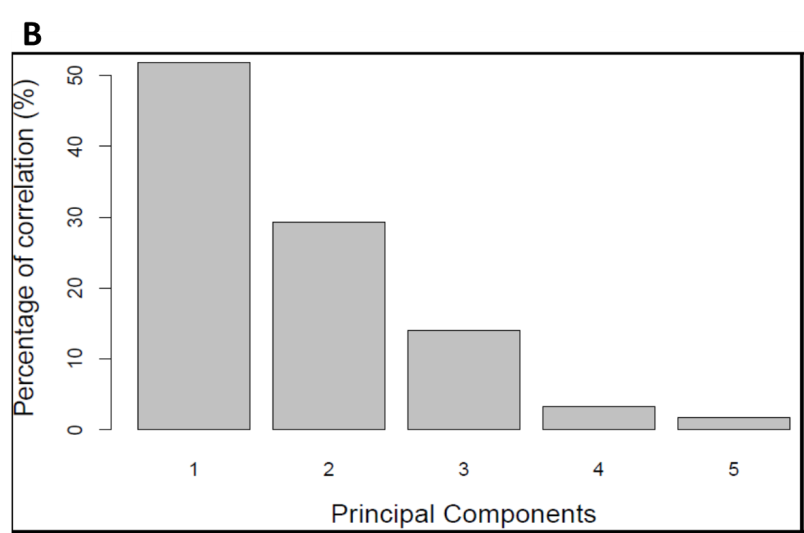
**

**Figure S5. Principal Component Analysis (PCA). A)** Biplot shows the result of a PCA of the correlation matrix of the interannual variability of MB, SF, SW, T, and LHF. The first two components explain 81% of the correlations. **B)** A screeplot showing the percentage of correlation accounted for by the principal components. In this analysis, we only consider the variables MB, SF, SW, T, and LHF. We exclude LW and SHF due to their strong correlations with SW and LHF respectively (Fig 4B). Similarly, albedo and TCC have strong correlations with SF and, therefore, are neglected. The first two principal components (Dim1 and Dim2) explain most of the correlations among the variables. SF (LHF) is strongly correlated with F1 (F2), with little correlation with F2 (F1). MB and SW have correlations with both the principal components, but a stronger overlap with F1. Thus, F1 signifies a strong control of SF fluctuations on MB variability, and on the corresponding variability of SW through albedo feedback.


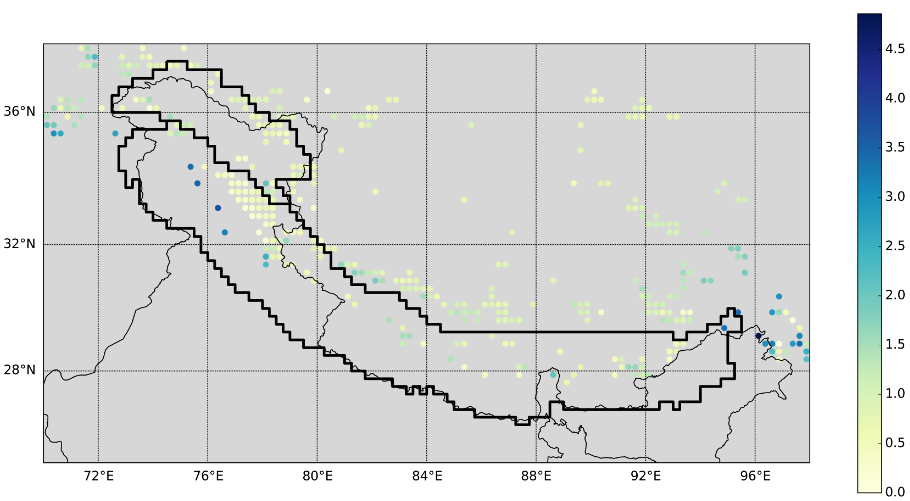


**Figure S6:** SF with region having glacier fraction less than 10%


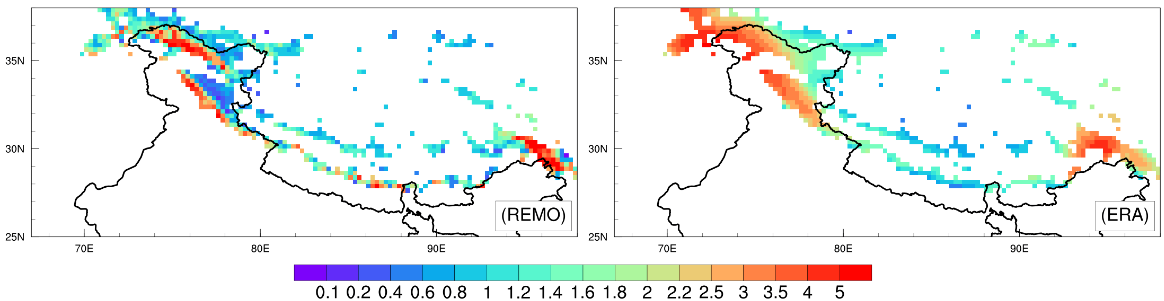


**B**

**A**


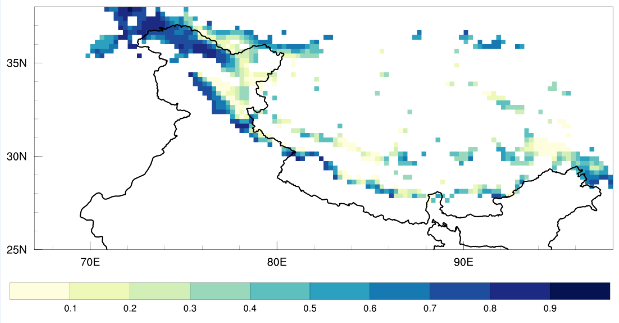


**Figure S7: Model and Reanalysis (ERAI) SF relationship, A)** SF simulated by modelled (upper-left) and ERAI (upper-right) (mm/day), **B)** Spatial correlation of model and ERAI snowfall. Dataset is plotted over glaciated fraction of gridbox only**.**

| 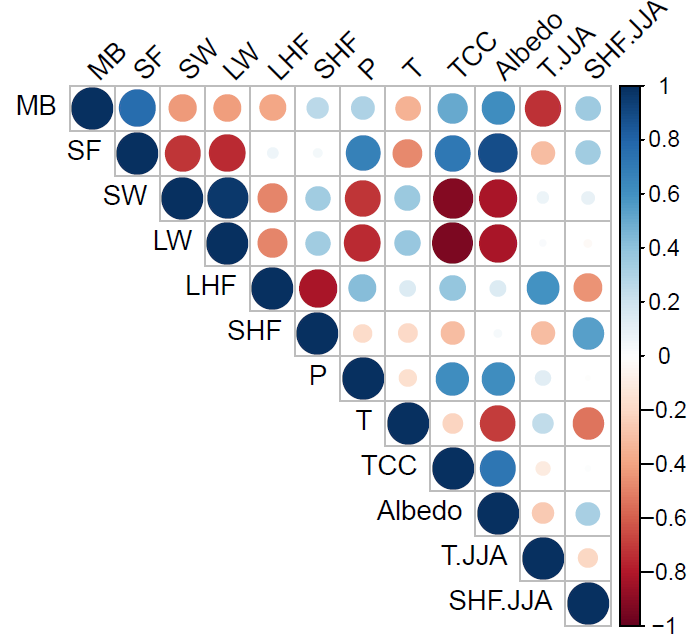  **Figure S8:** A graphical representation of correlations among the drivers of interannual variability of glacier MB in the HK along with summer temperature and sensible heat flux during 1990-2016.   \| 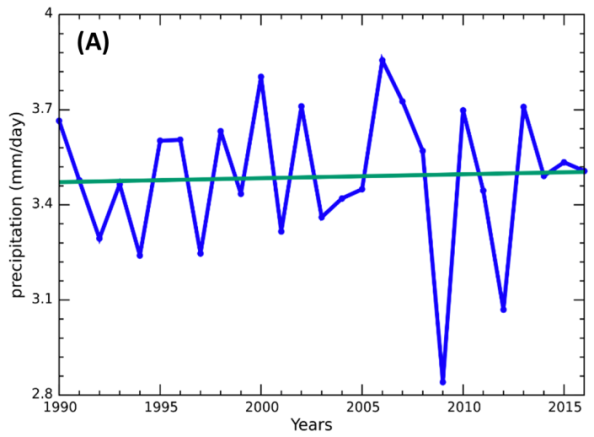 \| 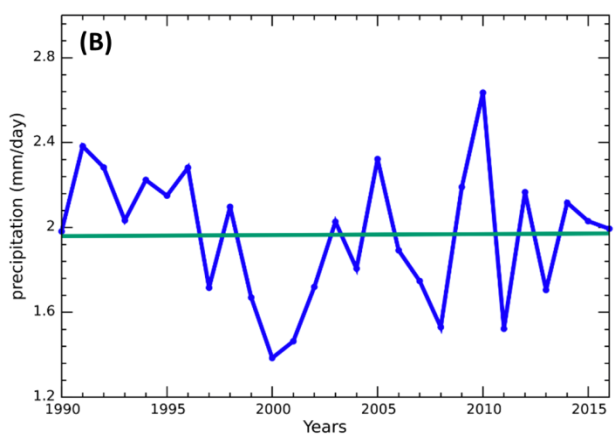 \| \| --- \| --- \| \| 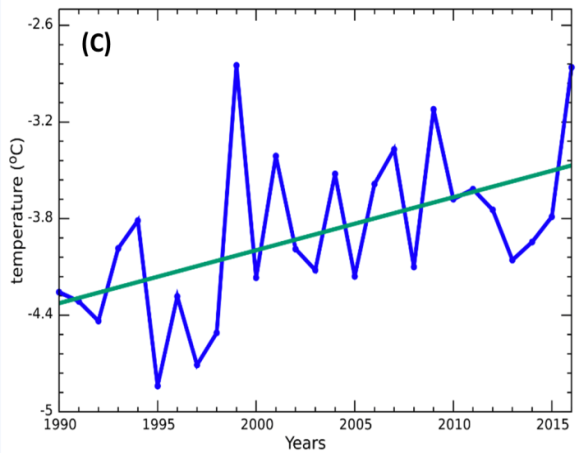 \| 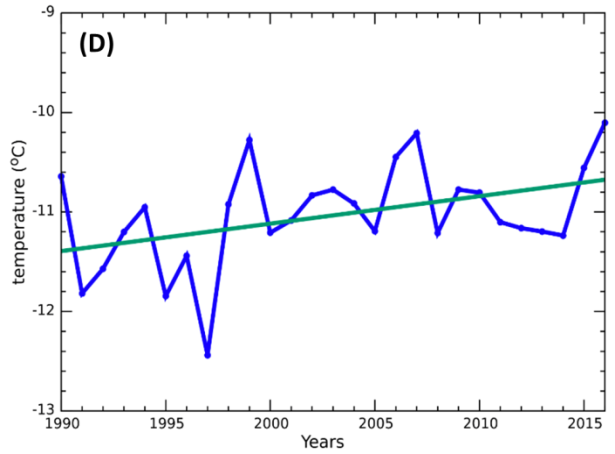 \| \| 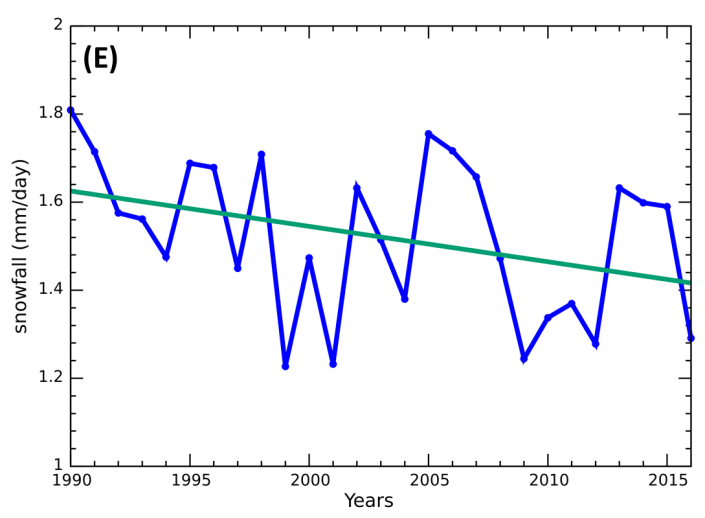 \| 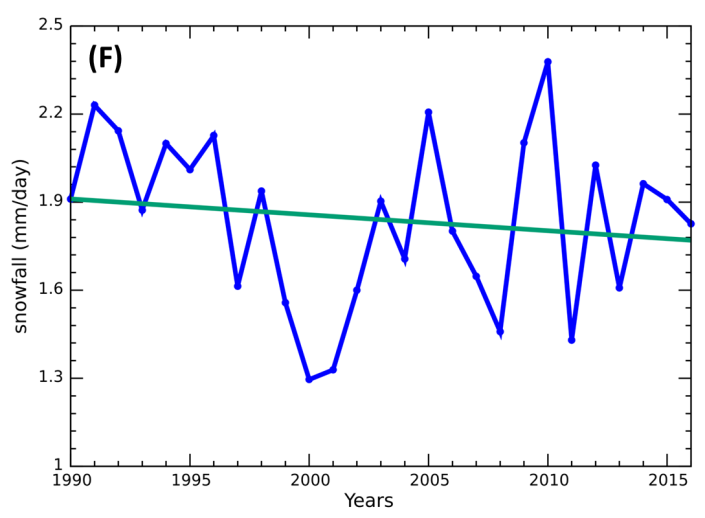 \| |  |
| --- | --- | --- | --- | --- | --- | --- | --- |
|  |  |

**Figure S9. Trends of P and T and SF over the Himalaya and Karakoram.** Annual precipitation in the Himalaya **(A)** and Karakoram **(B).** Annual temperature in the Himalaya **(C)** and Karakoram **(D)**. Annual snowfall in the Himalaya **(E)** and Karakoram **(F)**. Corresponding best-fit trend lines are also shown. The temperature trend is significant over Karakoram (p< 0.1) and H (p<0.01) region. The temperature trend in Himalaya and Karakoram is 0.32 ^0^C per decade (increasing) and 0.27 ^0^C per decade (increasing), respectively. The SF trend is significant over H (p< 0.1) only, while over K it is not significant. The snowfall trend in Himalaya is 0.08 mm/day per decade (decreasing).


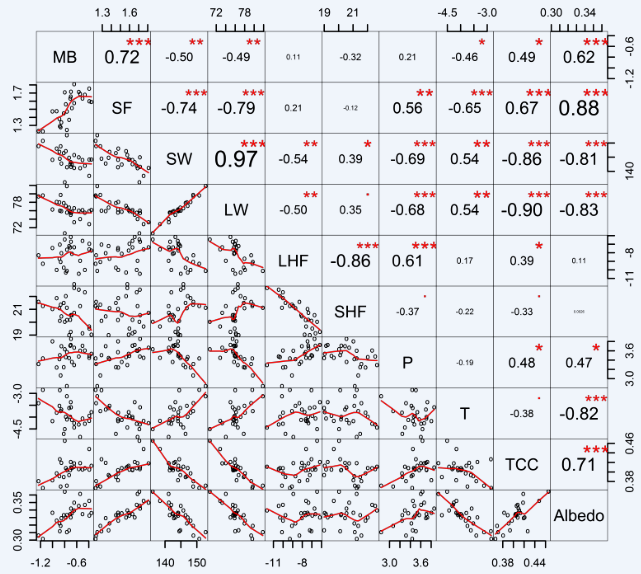


**Figure S10. Correlation Matrix.** The correlation matrix for MB and its drivers over the Himalaya. All conventions are the same as supplementary Fig S3.


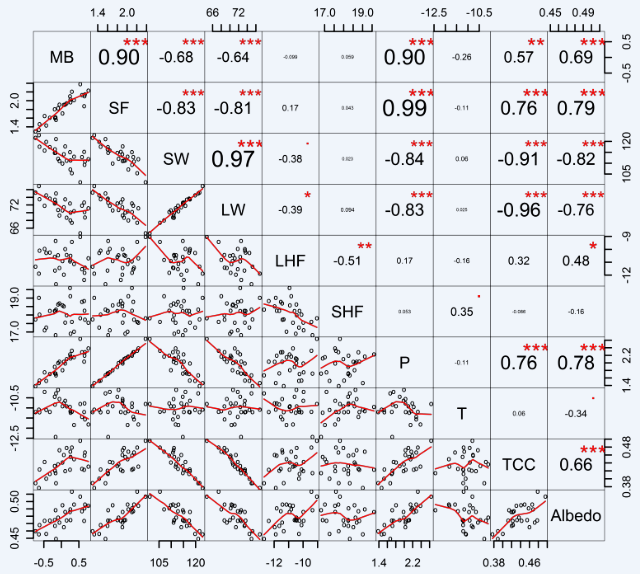


**Figure S11. Correlation Matrix.** The correlation matrix for MB and its drivers over the Karakoram. All conventions are the same as supplementary Fig S3.

**Table S1.** MB over FB (Brun et al., 2017) region for both modelled and FB data with their differences for the period 2000-2016.

| **MB** | **East Nepal** | **Hindukush** | **Nyainqentanglha** | **Bhutan** | **Pamir** | **Karakoram** | **Kunlun** | **Lahaul Spiti** | **West Nepal** |
| --- | --- | --- | --- | --- | --- | --- | --- | --- | --- |
| **FB** | -0.33 | -0.12 | -0.62 | -0.42 | -0.08 | -0.03 | 0.14 | -0.37 | -0.34 |
| **REMO** | -0.43 | -0.76 | -0.53 | -1.33 | -0.80 | 0.01 | -0.48 | -0.53 | -0.96 |
| **Diff.** | 0.10 | 0.64 | 0.09 | 0.91 | 0.72 | 0.04 | 0.66 | 0.16 | 0.62 |

**Table S2.** Modelled MBs value over HK, Himalaya and Karakoram regions for the period 1989-2016.

| **Years** | **Glacier Mass Balance (MB)- (m.w.e./yr)** | | |
| --- | --- | --- | --- |
|  | **HK** | **Himalaya** | **Karakoram** |
| 1989 | -0.393 | -1.003 | 0.137 |
| 1990 | -0.240 | -0.642 | 0.110 |
| 1991 | 0.149 | -0.566 | 0.771 |
| 1992 | 0.061 | -0.642 | 0.673 |
| 1993 | -0.016 | -0.781 | 0.648 |
| 1994 | -0.302 | -0.932 | 0.246 |
| 1995 | -0.060 | -0.544 | 0.361 |
| 1996 | 0.099 | -0.465 | 0.589 |
| 1997 | -0.466 | -0.814 | -0.164 |
| 1998 | -0.291 | -0.798 | 0.149 |
| 1999 | -0.622 | -1.162 | -0.153 |
| 2000 | -0.861 | -1.003 | -0.737 |
| 2001 | -0.916 | -1.233 | -0.640 |
| 2002 | -0.427 | -0.707 | -0.183 |
| 2003 | -0.279 | -0.680 | 0.069 |
| 2004 | -0.394 | -0.886 | 0.033 |
| 2005 | -0.022 | -0.393 | 0.300 |
| 2006 | -0.425 | -0.696 | -0.191 |
| 2006 | -0.580 | -0.699 | -0.477 |
| 2008 | -0.688 | -0.762 | -0.624 |
| 2009 | -0.001 | -0.764 | 0.662 |
| 2010 | -0.109 | -0.853 | 0.538 |
| 2011 | -0.569 | -0.726 | -0.433 |
| 2012 | -0.265 | -0.866 | 0.257 |
| 2013 | -0.410 | -0.503 | -0.328 |
| 2014 | -0.041 | -0.391 | 0.263 |
| 2015 | -0.195 | -0.356 | -0.055 |
| 2016 | -0.459 | -0.879 | -0.095 |
| **Mean** | **-0.311** | **-0.740** | **0.06** |
